# Promoting Safer Sexual Behaviours by Employing Social Cognitive Theory Among Gay University Students: A Pilot Study of A Peer Modelling Programme

**DOI:** 10.3390/ijerph17051804

**Published:** 2020-03-10

**Authors:** Luis Miguel Dos Santos

**Affiliations:** Woosong Language Institute, Woosong University, Daejeon 34514, Korea; luismigueldossantos@yahoo.com; Tel.: +82-010-3066-7818

**Keywords:** gay undergraduate, LGBT, Peer Modelling Programme, sexual behaviours, sexual education, sexual health promotions, sexual minorities, social cognitive theory

## Abstract

Unsafe and unprotected sexual behaviours are some of the significant challenges of health promotions and planning for current school environment. Although schools and health specialists constantly host conferences and workshops for adolescents and university students, the results are not significant. Particularly for sexual minorities, the heterosexual-oriented materials may not satisfy their needs due to the differences. As a recommendation, the current pilot study established a Peer Modelling Programme which engaged gay social workers and gay university students who have associated with unsafe and unprotected sexual activities. The outcomes of this Peer Modelling Programme indicated that gay undergraduate students tended to accept the recommendations and peer modelling exchanges from their gay social workers who understood their difficulties and sexual needs as sexual minorities based on the guideline of Social Cognitive Theory. In conclusion, this study may be used to develop additional social work materials, sexual health promotions and health plans for sexual minorities and people with special needs in the society. This research serves as a guideline to social workers who care about the issues of LGBT and sexual minorities.

## 1. Introduction

Protected and safer sexual behaviour, such as using a condom or having a single sexual partner or a stable relationship, are among the factors that protect individuals from sexually transmitted diseases (STDs), human immunodeficiency virus (HIV), and acquired immunodeficiency syndrome (AIDS) [1]. In Hong Kong, the Centre for Health Protection (2019) [2] reported that, on the basis of the accumulated statistics up to late 2019, 8306 males were infected with HIV and 1723 were infected with AIDS, while the corresponding figures for women were 1844 and 352, respectively; among those individuals, 4133 were infected by HIV due to homosexual contacts, while 3329 were infected due to heterosexual contacts. During the third quarter of 2019 (July to September), 61 individuals were infected with HIV due to homosexual contacts, while 38 individuals were infected due to heterosexual contacts. The trend indicates that homosexual individuals have a higher chance (almost 50% higher) of being infected than heterosexual individuals [2]. Although health specialists, teachers, and parents usually discourage underage and unsafe sexual behaviours among youths, one study of US college students indicated that 46% of them had engaged in sexual behaviours during high school and 38.9% of them had never used any form of protection, such as a condom [3]. Such unsafe sexual behaviours endanger the health of youths and teenagers, particularly sexual minorities such as lesbian, gay, bisexual, and transgender (LGBT) individuals [4]. Knowledge of safe and protected sexual behaviours is the key to protecting youths and sexual minorities from STDs infections. Regardless of whether it is based on theoretical knowledge, practical exercise, oral transfer, or peer modelling, correct knowledge on safe sexual behaviours is always beneficial [5].

Studies [6] have indicated that youths do not change their practical knowledge and practice after the completion of any conferences and lessons for theoretical knowledge of STDs, HIV, AIDS, and safe sexual behaviours. Although teachers, health specialists, and school nurses promote safer sexual behaviours via workshops, conferences, and lessons, youths usually do not gain the expected knowledge from these channels. Researchers [7] indicated that after health workshops, teenagers continue to engage in unsafe sexual behaviours as the lessons, which provide theoretical knowledge, do not increase their interest in safe sexual practices. Another study [8] indicated that although students usually understand the risks of unsafe sexual behaviours due to the knowledge they gained during their secondary education, they continue to engage in unsafe sexual behaviours as there is a gap in their knowledge concerning the use of protection techniques [9]. While scholars have advocated that knowledge of safe sexual behaviours may not increase individuals’ interest in using safer techniques when engaging in sexual behaviours, some reports have indicated that knowledge on condom use may reduce potentially unsafe sexual behaviours [10]. One study found that university students generally do not like to use condoms and other forms of protection as most believe that casual sexual behaviour between peers and classmates will not usually lead to the transmission of STDs due to the safe sexual behaviours and conduct of such individuals [11].

Previous studies have suggested various methodologies for sexual health promotions, including parental involvement and education, whereby parents act as role models and educators for safe and protected sexual behaviours [12]; workshops and lessons provided by school health specialists; and the mixed model (involving health knowledge, practical exercises, the behavioural skills model, and the social cognitive model) in which self-efficacy, modelling, social cognitive notions, and peer norms are utilised to promote the use of protection techniques and tools [13].

### 1.1. Background of This Pilot Study and the Peer Modelling Programme

In coordination with a group of social workers who care about the promotion of sexual health among LGBT individuals and sexual minorities in the Hong Kong Special Administrative Region (HKSAR), where the East Asian culture influences the social environment, the researcher established a *Peer Modelling Programme* (PMP) to provide “modelling education” to the targeted participants (i.e., gay undergraduate students).

This study was a pilot study about how the PMP can be contributed and beneficial to the field of social work. Before the researcher and the centres contributed additional resources, human resources, and patients into this PMP, the researcher tested the application and effectiveness of this PMP as a pilot study with a limited number of participants and social workers. As a result, the researchers received positive feedback about the effectiveness and results of this PMP from both social workers and participants.

The LGBT and sexual minorities’ issue is still considered as a taboo in HKSAR. The society still cannot accept same-sex relationship and same-sex marriage as a form of relationship. However, LGBT people and sexual minorities, and related issues are always found. Therefore, the researcher needs to develop an application to meet the special needs of these groups of people. The application of this study (i.e., PMP) can be further contributed to the special needs of single parents, disabled people, prisoners, and ex-mentally ill people who also demand special needs.

First, the researcher and four licensed social workers created a PMP with a focus on sexual health for gay men who have sex with men (MSM). Unlike other sexual health promotions and health plans (e.g., for heterosexual individuals), those targeted at MSM and their sexual behaviours may involve additional knowledge and addressing different sexual practices. Therefore, sexual health promotions and health plans for MSM have to be unique.

Second, to establish the notion of peer modelling, the social workers who participated in this study had to be gay men. Youths and adults usually receive theoretical and practical knowledge of health promotions and health plans through various channels. However, due to the cultural background of HKSAR, most of this knowledge concerns heterosexuals. In this study, there was common ground between the licensed social workers, all of whom had received their K-12 education and qualifying training in the HKSAR, and the participants because they were all victims of the incomprehensive nature of health promotions and health plans for sexual behaviour and thus fully understood this social issue.

Third, each social worker was paired individually with four gay undergraduate students. The social workers took on the role of peers and engaged in the participants’ daily life. They contacted each participant via social media chats every day (i.e., WhatsApp or WeChat) and a bi-weekly face-to-face café chat. Within the exchanges and conversations, the social workers contributed to sexual health promotions for MSM as peers and friends via the PMP. Details of the procedures and steps are explained in the methodology section.

### 1.2. Purposes of the Study

One major purpose formed the direction of this study. Safe sexual behaviours are the primary factors in preventing further cases of STDs, HIV, and AIDS among youths, teenagers, and adults [14]. Although the Department of Health and other NGOs continue to promote the importance of safe sexual behaviours internationally, the number of infected individuals continues to increase, particularly among gay individuals. HKSAR residents’ knowledge of sexually transmitted diseases is insufficient [15]. Therefore, the current study collected feedback about the effectiveness of this PMP from the perspective of social workers who have provided counselling to the participants (i.e., gay undergraduate students) [16]. Based on the feedback of the social workers, the researcher would like to understand the effectiveness of this PMP (i.e., pilot study) and how to develop this PMP into a larger programme for additional minorities with special backgrounds [17]. The current pilot study only involved a small population. If the result of this PMP (i.e., pilot study) was positive, a further and larger study would be conducted based on the results and recommendations.

### 1.3. The Theoretical Framework and Its Application

To understand how to effectively promote sexual health and safe sexual behaviour among youths and undergraduate students, particularly gay university studies in HKSAR, the researcher employed social cognitive theory [18].

**Figure 1:**
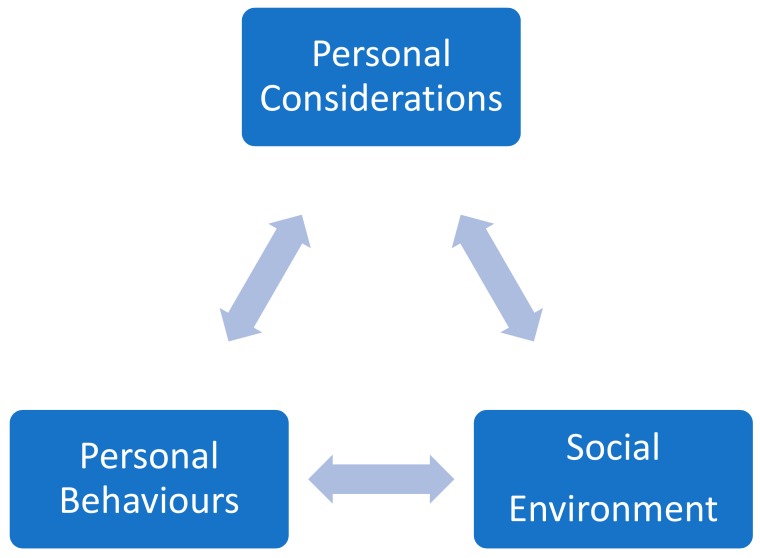
Bandura’s Triadic Reciprocal Causation Model [21].

Social cognitive theory suggests a triadic relationship between the individual, the individual’s personal behaviours, and external factors, and it is used to understand sexual behaviours among people [19]. Established during the 1980s, social cognitive theory advocates that people’s behaviours are guided by purposes and goals that are motivated via their personal beliefs of self-efficacy and by goal expectations from their behaviours within a particular social environment. Employing a three-directional model called the triadic reciprocal causation model [19], social cognitive theory describes individuals through the interconnections of three components (the individual, the individual’s behaviour, and the environment) that influence one other. Bandura’s [19] triadic reciprocal causation model suggests that individuals’ behaviours and choices usually interact with and influence each other. The personal considerations component of the model involves cognitive, affective, and biological considerations. The social environment component involves the surrounding factors, such as cultural and political impacts. The personal behaviours component involves how individuals react to specific events and circumstances. Figure 1 provides an outline of Bandura’s triadic reciprocal causation model [19]. More recently, Bandura [20] further explained that within the interactions and connections between an individual and the social environment, internal personal factors in the form of cognitive, affective, and biological events; personal behaviours; and environmental events all operate as interacting determinants that impact each other bi-directionally [21].

Within the personal considerations component of the triadic reciprocal causation model, Bandura suggests three factors which may greatly influence individuals: self-efficacy beliefs, outcome expectations and goals, and self-regulated learning [21]. Individuals’ behaviours are significantly influenced by self-beliefs and understanding. Thus, social environmental features only impact a certain amount of personal behaviours.

In the field of health science and school well-being, social cognitive theory has been employed to understand the behaviours associated with alcohol use, obesity, school bullying, adolescent smoking habits, and drug use; for example, one study [22] indicated that social cognitive theory has positive influences and impacts on strategies to promote correct alcohol and drinking behaviour and to provide plans for youths and adults who are addicted to heavy alcohol use. Factors such as family relationships (siblings, parents), personal characteristics, cultural background, and even religious concepts may influence youths’ and adolescents’ views on alcohol and drinking. One study [23] on drug and substance abuse among adolescents found that youths tend to (a) model their behaviours on those they observe in the people around them and (b) be influenced by external and environmental factors; for example, family management and demographic and socioeconomic characteristics can greatly influence adolescents’ views on substance and drug use. Although most of the youths in that study understood that using drugs harmed their health, most believed that external and environmental factors encouraged their consumption behaviours.

Recently, a study [24] advocated that college peer health education groups provided by university departments and counselling centres may serve as a model and channel to promote health and wellness information to university-level students on campus. The study indicated that peer education and modelling were effective in increasing knowledge and understanding of sexual health and protected sexual behaviours (e.g., using a condom). Another study [25] collected data from 300 early adolescents on how their sexual attitudes were influenced by the use of internet chatrooms and peer-to-peer exchanges. The results showed that compared with their pretest responses, 78% of the youth participants provided riskier responses during exchanges with peers via the chatroom environment. The results also indicated that male adolescents are more likely than girl adolescents to be influenced by peers and unknown internet friends and peers. Although studies have been conducted on sexual behaviours among youths and adolescents from the perspective of social cognitive theory, none of these studies explored the issues for LGBT individuals and sexual minorities, particularly in the East Asian context.

## 2. Materials and Methods

### 2.1. Participants

**Figure 2:**
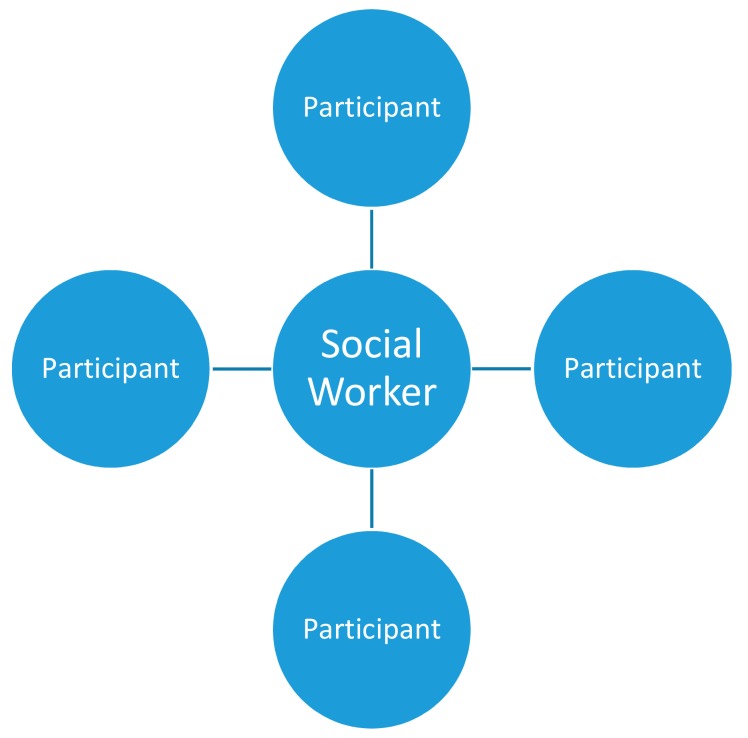
The relationship between social workers and participants.

Four licensed social workers and 16 gay undergraduate students in the HKSAR were invited. First, due to the nature of the PMP, the social workers and participants had to be gay men. Second, the participants had to have previous experience of receiving sexual health promotions. Third, regardless of nationality, the participants had to be current undergraduate students in the HKSAR. Fourth, the participants had to be at least 18 years old and willing to participate. As a result, 16 gay undergraduate students ranging in age from 18 to 24 agreed to participate. After the matching procedure, each social worker was paired with four gay undergraduate students individually. Figure 2 indicated the relationship between the social worker and the participants. Each participant does not have any connections with each other. The social worker coordinated and provided peer modelling counselling to each participant.

The researcher did not participate in the counselling sessions as the researcher is not a licensed social worker. The researcher’s role was to develop and contribute ideas and potential strategies which could help the social workers. In other words, the researcher worked as an administrator for the developing stage and data collection and analysis.

### 2.2. Procedure of the Peer Modelling Programme

#### 2.2.1. Pre-Peer Modelling Programme Chat

A social worker paired up with a participant in a peer modelling group. Both met in a private room where they shared their sexual behaviours, sexual history, and knowledge of sexual health, protection plans for homosexual behaviours, and MSM. The social worker recorded the information shared by the participant for further development (i.e., self-created checklist). Before the end of the chat, the social worker and the participant established a social media chat room for exchanging information and making further appointments for their bi-weekly chats.

#### 2.2.2. The Six-Month Peer Modelling Programme

As sexual behaviours and attitudes may not be easy to change due to cultural and social background, to increase the effectiveness of the PMP, both had to participate for at least six months.

During the six-month PMP, the social workers were allowed to ask open-ended and semi-structured questions based on the progress. Each participant was provided with a portfolio of their progress (e.g., understanding of sexual behaviours, number of unprotected and protected sexual behaviours, condom use, sexual partners, etc.). The social workers were required to indicate the progress of each participant’s individual procedures of their sexual knowledge, particularly with regard to MSM.

The counselling materials were designed by social workers. Each participant has a unique background. The social workers have their unique materials, procedures, strategies, and counselling methods based on the needs of their patients. Also, as this PMP focused on the sexual health promotions between both gay individuals, the social workers should adjust the pre-set materials and strategies based on the needs and sexual behaviours of their participants. Therefore, the researcher provided the freedom to the social workers for their professional counselling and PMP arrangements.

#### 2.2.3. Post Peer Modelling Programme

After the six-month PMP, the social workers chatted with the participants individually about their development and progress with regard to sexual health behaviours and knowledge. The social workers reviewed each participant’s portfolio, and the answers from the self-created checklist. The social workers discussed the checklist knowledge and indicated the behavioural and knowledge changes after completing the PMP.

#### 2.2.4. Post Peer Modelling Programme Interview with the Researcher

To gain the answer to the purpose and to assess the effectiveness of the PMP, the researcher invited each social worker for an individual semi-structured interview to give their feedback and recommendations on the PMP. Each individual interview lasted between 90 and 134 min due to the in-depth exchanging. Besides, the researcher invited four social workers to a focus group activity for group exchanging. The focus group activity lasted 123 min.

#### 2.2.5. Member Checking

To increase the validity of the study, the researcher sent the analysed data to each social worker for confirmation. Also, the follow-up member checking interviews were conducted to verify the data. Each member checking interview session hosted between 45 and 68 min.

### 2.3. Data Analysis

All conversations were digitally recorded, transcribed, and returned (i.e., member checking) to the social workers individually for validation of the content. Once the social workers approved their own transcripts and conversations, the data were analysed.

Interview and focus group activity data were the key components of the data analysis. Themes were categorised. The general inductive approach [26] was employed to narrow down the large-size transcripts (208 pages) into first-level themes using the open-coding technique from the perspective of the grounded theory approach [27]. Qualitative researchers advocate that for large-size transcripts, a researcher should read through information at least five times and begin to categorise general directions. In this study, 21 themes and 25 subthemes were identified based on the first-level coding technique.

The general inductive approach advocates that data information should be narrowed down for further reporting. Therefore, the axial-coding technique was employed to reduce the data information into second-level themes. Axial coding involves categorising the relationships among all of the open-coding results. In this study, the researcher eventually narrowed the data down to three themes and three subthemes for reporting.

### 2.4. Human Protection and Ethical Consideration

The protection of human subjects was important, particularly given the study’s focus. Therefore, the researcher made every effort to protect the identities, allowing them to remain anonymous to any parties. Also, in the report, each person was identified solely by their role (e.g., Social Worker #1; Participant #2). All subjects gave their informed consent for inclusion before they participated in the study. The study was conducted in accordance with the Declaration of Helsinki, and the protocol was approved by the Ethics Committee of The Social Caring Centre for HIV/AIDS (2019/Summer/Fall/1123).

### 2.5. Limitation of the Participants

A limitation of this pilot study was the recruitment of participants. In this pilot study, the researcher only invited four gay social workers and 16 gay undergraduate students as the participants. The current database did not indicate the sexual orientation of the social workers and participants. Therefore, based on personal networking of the researcher, the current pilot study could only invite the abovementioned people. After the completion of this pilot study, the researcher may employ this pilot study and its directions into larger-size research.

## 3. Results

During each interview section, the social workers answered the same semi-structured questions about the PMP. Although the social workers were paired up with the same number of participants with a similar background and sexual orientation (i.e., one social worker with four participants), their experiences and progress were not the same. The issue of LGBT and sexual minorities is still a social taboo in HKSAR due to the East Asian cultural perspective that prevails in the region. Although the PMP matched the social workers and participants with the aim of producing peer relationships, building solid relationships to facilitate the exchange of lived experiences takes time. Therefore, the PMP ran for six months to enable trust and understanding to be developed.

During the data collection sessions, the social workers stated that the PMP is an excellent technique to increase the overall knowledge and practical skills of gay undergraduate students. None expressed negative opinions about the PMP. The findings were categorised into three themes and three subthemes. The details have been listed in Table 1.

### 3.1. Successfully Built Up a Long-Term Relationship with the Gay Communities

Peer modelling is one of the significances of social cognitive theory. In accordance with social cognitive theory, the PMP established the pairing relationship between the social workers and the participants to build up the peer modelling role. Before the pairing procedure, none of the social workers or participants knew each other. Although the social workers stated that they usually hosted passive workshops for sexual minorities, only a few participated in these meetings actively. The pre-set workshops and conferences were pre-created with general knowledge which may not meet the needs of sexual minorities. However, the current PMP was individually created based on the needs of each participant. Therefore, the social workers received better and more accurate information from the gay undergraduate student communities and other LGBT groups.

#### 3.1.1. Improved and Polished In-Depth Sexual Health Promotions and Health Plans

The PMP allowed the social workers to improve and polish the current homosexual and heterosexual sexual health promotions based on the portfolios and progress. A social worker stated that after the completion, he received useful information to fill the knowledge and practice gaps in current sexual health promotions:

*…the current sexual health promotions s were written by heterosexual individuals who might not have any knowledge of homosexual behaviours…That knowledge is outdated, not useful, and non-practical due to the misunderstanding of and social bias against LGBT and sexual minorities…* (Social Worker #1, Interview)

The concern about outdated and non-practical information. Sexual minorities may be involved in same-sex behaviours. Some youths may believe that same-sex behaviours cannot transmit any diseases. Social Worker #2 indicated that if there are no further improvements in the current sexual health promotions, the next generations will continue to engage in unsafe sexual behaviours:

*…the lessons and materials never touch the issues of same-sex behaviours and unprotected sexual intercourse between men…between women…or perhaps transgender individuals…some youths may have the misunderstanding that MSM behaviours are safe as no vaginal intercourse occurs…* (Social Worker #2, Interview)

In addition to stressing the benefits of the PMP in terms of improving sexual knowledge, the social workers advocated that adults, parents, school teachers, health specialists, and nurses could benefit. Due to social developments, different types of relationships, sexual behaviour, and internet chat groups have emerged. The materials of sexual health promotions, however, have not been updated appropriately. Social Worker #4 described how one man told him that got involved with group sex activities using only one condom:

*…some men believe that they only need to protect their body but not others…A peer told that he only used a condom during group sexual intercourse with four other people …However, he used that single condom to engage in sexual intercourse with the other four …He can protect himself…but transmit diseases to other members of the group…* (Social Worker #4, Interview)

Social Worker #3 stated that in addition to providing insights into the various types of sexual behaviour practised, the PMP and the sharing of information from his clients had increased his knowledge of how to promote sexual health to all who live under the East Asian culture:

*…Oral sex is not uncommon…but the sexual health materials always ignore this…as well as anal sex…sexual behaviours do not only occur among LGBT individuals and sexual minorities…heterosexual individuals engage in these behaviours and even transmit STDs and HIV via unprotected intercourse…At the peer level via this PMP, I gained frontline information from people who experience these behaviours almost weekly…* (Social Worker #3, Interview)

Based on the developments from the data, it indicated that peer modelling and information from peers could influence the decision of youth based on the guideline of social cognitive theory [19]. The social workers expressed that the exchange of information at the peer-level via the PMP allowed them to gain information from their clients. Instead of creating materials and polishing the information without feedback from the users, the peer relationship with clients allowed them to enter the inner world of their clients and understand how they made sense of their lived experience as gay undergraduate students.

#### 3.1.2. Individualised Needs: Social Bias and Taboos

All advocated that heterosexual and homosexual behaviours and attitudes are not the same due to physical and mental differences. The current sexual health promotions for heterosexual individuals do not meet the needs of any LGBT individuals or sexual minorities.

By combining the social workers’ homosexual experiences and the experiences shared by their clients, the peer modelling relationships and needs of LGBT and sexual minorities can be established based on the social cognitive theory approach [19]. All expressed that by combining their lived experiences and the experiences their clients shared, they could formulate better sexual health promotions for both LGBT and heterosexual individuals. It reflected how peer modelling and sharing could be influenced by the decision of individuals, saying:

*…homosexual and transgender individuals do not need to care about pregnancy…but the sexual promotion materials always concern pregnancy and single mothers…for gay individuals and their sexual health materials, we need to focus on STDs, HIV, and other illnesses instead of something that never happens…* (Social Worker #4, Focus Group Activity)

On the other hand, all expressed that young gay undergraduate students predominantly date other gay men via social media platforms and cell-phone applications. Instead of ignoring the fact that youth like to have sex with others, social workers should guide gay individuals to protect themselves. All indicated that sexual health promotions should focus on internet dating and even one-night stands. A social worker believed that materials for youth should focus on online dating and sexual behaviours:

*…many young gay students and individuals like to date online friends made using cell-phone applications…they don’t know each other but are willing to engage in sexual behaviour and even unprotected intercourse …The current outdated materials in the library cannot respond to the problems…* (Social Worker #3, Focus Group Activity)

Another social worker stated that internet dating has become very popular among gay youths recently. Individualised instructions from the peer level would be useful. A social worker used his own lived experience to share ideas on sexual health as a gay peer via the PMP:

*…gay men can influence another gay man…heterosexual individuals usually cannot understand the behaviours, ideas, and thinking of the LGBT and sexual minority communities…So I can use my peer and lived experience…from this PMP …to influence other groups of gay youths…* (Social Worker #1, Interview)

#### 3.1.3. Successful Referral of other Gay Individuals

All indicated that during the six-month PMP, their clients referred other sexual minority peers and friends for information regarding sexual health, particularly HIV testing. Although the current PMP involved 16 participants, the social workers can employ the programme in their daily casework. Surprisingly, all stated that the PMP allowed them to “gain an in-depth understanding of and make friendships within the gay communities” (Social Worker #2, Interview). One social worker indicated that the PMP promotes “peer-level exchanging and story-telling techniques that increase the trust and relationship between gay peers” (Social Worker #1, Focus Group Activity).

All believed that the nature of the PMP allowed them to establish a peer-level relationship, friendship, and peer modelling roles [19] with their clients instead of a top-down relationship. Youth tend to listen to their peers instead of teachers, leaders, and parents as described by Social Cognitive Theory and reflected a previous study about how students reacted to their self-efficacy and decision making process [1]. Therefore, youth would be willing to refer other peers with similar backgrounds to social workers who they regard as their peers. As one social worker said:

*…my client and I shared a similar background and the same sexual orientation, and so we could have a deeper conversation about some topics…[A]s we chatted via WhatsApp and met bi-weekly over the months, we built up a friendship instead of a client relationship…my client sometimes referred some gay friends to me for HIV testing…once I have the new client’s contact details, I start to help others…* (Social Worker #4, Interview)

### 3.2. Successful Increased Sexual Health Knowledge and Practice: External Influence

All in this study stated that the PMP allowed them to provide additional information about sexual health practice to youth and young gay undergraduate students as peers and friends. A study indicated that gay individuals tended to believe their peers for sexual recommendations [14]. One social worker expressed that regular HIV testing can be a health protection measure for all individuals in society:

*…in general, the public believe that HIV testing is a dirty test for sex workers…but this test can be taken by all engaged in any sexual behaviour…the test allows clients to understand the condition of their health…having this test is positive sexual health practice…* (Social Worker #4, Focus Group Activity)

During the focus group activity, social workers also expressed that teaching clients to avoid drug and alcohol misuse is important. Sexual abuse and sexual violence are different types of unsafe sexual behaviour that many people do not pay attention to, particularly youths and young LGBT and sexual minority individuals. The social workers expressed that several of their clients had experienced unwilling sex but did not know how to refuse, “…many youth did not know how to refuse unwilling and unprotected sex with their partners…or even during group sex …although such types of abuse are illegal, they do not understand how to refuse sex…” (Social Worker #1, Focus Group). Another social worker expressed similar ideas about how refusing sexual abuse can be harmful to sexual minorities:

*…many youth don’t understand that they can refuse unwilling and unprotected sex …some even believe they have to suffer pain to get appreciation from others…some even told me that they want to join the community, so they need to have sex with some people…we have to tell them these ideas are wrong… (Social Worker #3, Focus Group)*

### 3.3. Successful Improved Sexual Behaviours and Use of Protection: Internal Influence

Besides the external and environmental influences, the social workers shared their lived stories and experiences to stress guidelines to youth on the importance of safe sexual behaviours and knowledge at the peer level. The second theme indicated that the social workers transferred essential knowledge on topics such as physical protection from sexual abuse; drug use; condom use; and regular HIV testing. This theme also indicated that social workers believe that mental and internal influences are important.

From their experience of the PMP, the social workers stated that before their clients participated in the PMP, most had had at least five sexual partners and had never experienced protected sexual behaviours. The numbers are significant as they indicate that many young gay individuals have been putting themselves in danger:

*…[Having a] single sexual partner can effectively reduce the potential transmission of STDs and HIV…although we cannot prevent our peers from having sex with others, we need to tell them to use a condom at least…but I or we must try our best to teach them the notion of having a single sexual partner…* (Social Worker #3, Focus Group)

Furthermore, all indicated that multiple sexual partners and excessive sexual behaviours are not uncommon among heterosexual, LGBT, and sexual minority communities. However, social media and expectations have stigmatised and created a bias against the sexual minority communities. The previous study indicated that social bias and social cognitive approach about multiple and unsafe sexual behaviours always toward sexual minorities. As a result, some youth believe having multiple sexual partners and engaging in excessive sexual behaviours are expected [6].

Based on the peer modelling approach from social cognitive theory [6,14,19], the social workers stated that they had to spend months correcting such inappropriate ideas from their clients. One commented how *“two peers within the PMP believed they should have multiple sexual partners at one time to show their adulthood”* (Social Worker #1, Interview), while another stated that some gay undergraduate students believed that *“without multiple sexual partners, [they] could not show their adulthood…as a man”* (Social Worker #2, Interview).

Fortunately, most of their clients changed their notions about multiple sexual partners and excessive sexual behaviours after the completion of the PMP due to the peer-level exchanges and sharing of lived stories during the programme [6,14,19]. One social worker expressed that most youths do not listen to social workers:

[Social workers] can only share top-down directed knowledge and non-practical skills in lessons, conferences, or workshops…the youth tends to absorb knowledge from the internet, peers, and friends…social workers should change the ways they contribute to meet the needs of these groups of vulnerable individuals… (Social Worker #3, Interview)

## 4. Discussion

Sexual health promotions are significantly important in the areas of well-being, social welfare, health sciences, LGBT, and sexual minorities. According to the results, both four social workers and 16 gay undergraduate students had already participated in and completed the six-month PMP before the data collection procedure. Although the researcher did not invite gay undergraduate students due to the registration issues, the data were still meaningful. Most of the feedback from the social workers were positive and supportive. All social workers indicated that *“this is the first time I engaged with some sexual minorities and gay patients as a gay social worker, we can exchange our real difficulties and discriminations”* (Social Worker #1, Focus Group Activity). Without a doubt, the sexual orientation and the status of sexual minorities between the social workers and participants highly increased the level of trusts and effectiveness of the PMP. According to social cognitive theory [21], individuals’ behaviours can be influenced by personal considerations, personal behaviours, and social-environmental impacts. The results of this study discovered that the PMP is an excellent strategy to encourage gay undergraduate students with prior experience of unsafe sexual behaviours and intercourses from the peer-influences of other gay social workers.

This indicated that PMP engaging with social cognitive theory [21] is an effective theory in promoting safer sexual behaviours. This study gave credits to the issue that personal considerations, personal behaviours, and social-environmental impacts alongside with peer modelling are directly associated with safe and protected sexual behaviours and related to the earlier research which indicated the direct association between how peer modelling can impact individuals’ behaviours in the field of sexual promotion [6,14,19]. The study added to the previous literature reviews. However, there are only a few studies about gay undergraduate students. Based on the feedback, all social workers advocated that the current one-on-one relationship between their patients highly increased their sexual health knowledge. The current one-on-one PMP provided the individualised counselling strategies which matched the unique needs and behaviours. The social workers believed their peer-roles and recommendations as gay individuals may influence the overall performance due to the ideas of modelling and social-environmental impacts from social cognitive theory guidelines [21].

The unique relationship between social workers and participants was supportive from the perspective of modelling and social-environmental impacts by social cognitive theory [21]. The sharing from the social workers indicated how the personal considerations and personal behaviours inter-influenced the decision-making process. For example, the social workers indicated that the participants’ behaviours were changed due to their peer modelling and personal behaviours as gay individuals. Social cognitive theory [21] advocated that individuals’ behaviours would be changed due to peer modelling and sharing from their friends and peers in the social environment. Since this was a pilot study with limitation population, larger population from different backgrounds cannot participate in this study. To establish this relationship and study, there is a need for longitudinal studies in the future with similar individuals.

Earlier studies [1] indicated in a target group of minority university students found social cognitive approach and peer modelling toward safe and protected sexual behaviours was the significant predictor of their decision of sexual behaviours. This study has further expanded the guideline from social cognitive theory [6,14,19], the PMP was a very supportive channel to influence the sexual behaviours and increase the safer sexual behaviours of LGBT and sexual minorities, particularly gay undergraduate students. The social environment element from social cognitive theory [21] indicated that individuals were more likely to be influenced by the external environmental factors and influences from peers (e.g., peer modelling). In this case, the gay social workers served as the social environmental factors and peers to influence youth’s sexual behaviours (i.e., from unsafe to safer sexual behaviours). To be particular, the Triadic Reciprocal Causation [19,21] about the individuals’ sexual decision can be influenced by their peers and peer modelling strategies. Similarly, the personal considerations, personal behaviours, social-environmental, and alongside with peer modelling were found to be the significant influences of safe and protected sexual behaviour.

## 5. Conclusions and Future Directions

There were two limitations in this study. The population was limited to only four social workers and 16 participants. Due to the nature of the pilot study and limited populations of gay social workers and gay undergraduate students under the current human resource database, the researcher could only invite a small population in the study. After the completion of this pilot study, the researcher can further develop and invite additional minorities for a larger-size programme.

Besides, scholars may argue the researcher was the only person for data analysis. The objective of reporting may cause concern for the study. In order to overcome this concern, after the researcher completed the data analysis procedure, the researcher sent the data to each social worker for confirmation with a member checking interview session. For a larger-size study in the future, the researcher will invite a larger participants’ population and additional researchers into the study in order to increase the performance of the study.

This study contributes to the understanding of how social cognitive theory [6,14,19] could influence the sexual health promotions and health plans of LGBT and sexual minorities, particularly gay undergraduate students, via the PMP in the East Asian environment. First, the current PMP (i.e., pilot study) received positive feedback from the social workers and allowed the social workers to build up the peer modelling relationship based on the guidelines from the social cognitive theory [6,14,19]. Therefore, the successful data and results allowed the researcher expanding the PMP for additional minorities.

Second, the information from this study may be used to develop additional social work materials, sexual health promotions and health plans, safe sexual behaviours guidelines, and materials for LGBT and sexual minorities. Therefore, the current study and data information from social workers and participants always increase and enhance the details and knowledge of the current materials.

Last but not least, this study also provided the opportunities for Department of Health, Department of Education, Department of Social Welfare, government agencies, NGOs, school leaders, social workers, teachers, health specialists, nurses, and policymakers internationally to increase their understanding and knowledge about sexual health promotions and health plans for both heterosexual and homosexual individuals.

## Figures and Tables

**Table 1:** Themes and subthemes.

1. Successfully Built Up a Long-Term Relationship with the Gay Communities
1.1. Improved and Polished In-Depth Sexual Health Promotions and Health Plans
1.2. Individualised Needs: Social Bias and Taboos
1.3. Successful Referral of other Gay Individuals
2. Successfully Increased Sexual Health Knowledge and Practice: External Influence
3. Successfully Improved Sexual Behaviours and Use of Protection: Internal Influence
